# Nano-Metal Organic Framework for Enhanced Mechanical, Flame Retardant and Ultraviolet-Blue Light Shielding Properties of Transparent Cellulose-Based Bioplastics

**DOI:** 10.3390/polym13152433

**Published:** 2021-07-23

**Authors:** Lijian Sun, Limei Li, Xianhui An, Xueren Qian

**Affiliations:** Key Laboratory of Bio-Based Material Science & Technology Ministry of Education, Northeast Forestry University, Harbin 150040, China; lantian0308@nefu.edu.cn (L.S.); limeili937@foxmail.com (L.L.); anxianh509@163.com (X.A.)

**Keywords:** bioplastic, MIL-125(Ti)-NH_2_, fire-retardant, UV-blue light blocking, mechanical properties

## Abstract

From the perspective of sustainable development and practical applications, there has been a great need for the design of multifunctional transparent cellulose-based composite films. We herein propose a novel concept of improving the mechanical, fire-resistant and ultraviolet (UV)-blue light shielding properties of cellulose-based composite bioplastic films though in situ embedding nano-metal organic framework (MIL-125(Ti)-NH_2_) into regenerated cellulose gel. Regenerated cellulose hydrogel (CH) with a porous structure acts as a nanoreactor and stabilizer to facilitate the growth and anchorage of MIL-125(Ti)-NH_2_ nanoparticles (MNPs). Subsequently, hot-pressing induces the formation of transparent MIL-125(Ti)-NH_2_@cellulose bioplastics (MNP@CBPs). As expected, the MNP@CBPs exhibit exceptional UV-blue light shielding capability, while retaining satisfactory optical transmittance. Meanwhile, with the incorporation of MNPs, the mechanical strength of MNP@CBPs is increased by 6.5~25.9%. In addition, MNPs enhance the flame retardant effect of the MNP@CBPs. The limited oxygen index (LOI) of the MNP@CBPs increased from 21.95 to 27.01%. The hot-pressing process improves the resistance of the MNP@CBPs to the penetration of water/non-aqueous liquids. This simple strategy would direct sustainable multifunctional MNP@CBPs toward diversified applications: food containers or packaging materials that can reduce or eliminate food spoilage, screen protectors for blocking harmful light, and promising candidates for protective plastic products, among others.

## 1. Introduction

Growing environmental problems (e.g., microplastics) have dramatically highlighted the urgent need for fabricating sustainable polymers and polymer composites [1]. With regard to this, cellulose-based film materials have recently been developed and are increasingly replacing traditional fossil-fuel nonbiodegradable polymers widely used in the areas of battery separators [2], UV light-shielding [3], packaging [4], medical applications [5] and solar evaporation [6], etc., because of their high transparency, excellent flexibility, lower coefficient of thermal expansion, good mechanical strength, renewable and biodegradable features [7]. However, a neat cellulose film has limited properties, so there is an urgent need to develop new cellulose-based composite films with high mechanical strength and different functions to support advances in diverse strategic fields. In particular, neat cellulose films suffer from intrinsic high flammability and poor blocking of UV-blue light, which is viewed as a stumbling block which gravely restricts the development and application of cellulose films.

Among the advanced features, flame retardancy of cellulosic materials is highly desired. High flammability is an intrinsic characteristic of cellulose-based materials, and there is little residual char formation during combustion. So far, the incorporation of various flame-retardant materials, such as phosphoramidate siloxane polymer [8], magnesium hydroxide nanoplatelets [9], montmorillonite [10], sodium bicarbonate [11], and silica nanoparticles [12], has been one of the most widely studied methods to impart cellulose films/paper with fire-retardant properties. Meanwhile, some composite materials, such as lignin-modified carbon nanotube/grapheme hybrids, have been used as flame retardants [13]. Despite the significant improvement in flame retardancy, the resulting cellulose-based materials usually require a high content of flame retardants to obtain satisfactory flame-retardant effects, which in turn leads to a loss in the mechanical strength, flexibility and optical transmittance cellulose-films. Therefore, it is urgent to select suitable flame retardants to provide cellulose films with good flame retardancy, transparency and mechanical strength, especially non-toxic flame retardants.

In addition to the high flammability, the poor UV-blue light blocking capability of cellulose films due to the lack of UV-blue light-absorbing or -shielding structures hinders their application as protective materials such as electronic devices or food packaging [14]. The potential hazards of UV radiation on human health and material preservation have attracted widespread attention. Excessive exposure to UV radiation can cause many problems, such as skin burns, loss of mechanical strength and photodegradation of organic materials [15]. It is well known that blue light has a negative effect on the eyes, including optic nerve crush, accumulation of eye strain, photoreceptor cell destruction and macular degeneration [16]. Hence, the filtering of UV-blue light will become significant in the future. Currently, the general methods to obtain a UV-blue light shielding function is mainly by incorporating inorganic and/or organic UV-blue light absorbers into the cellulose matrix [17]. However, employment of these absorbers is accompanied by some serious problems represented in difficulties relating to the preparation process, which includes many steps/high chemical consumption, and using hazardous chemicals. There is therefore an urgent need to develop efficient UV-blue light shielding materials that can be synthesized through a simple strategy.

In order to achieve the above functions, the use of nanomaterials to modify and functionalize cellulose films has become a very effective method [18]. Although significant progress has been made in this field, it remains a challenge to simultaneously obtain multifunctional cellulose films with flame retardant, high mechanical strength, and UV-blue light shielding properties for practical applications. Thus, it is particularly meaningful to search for a kind of nanomaterial with potential flame retardancy, UV-blue light blocking and the potential to increase the strength for fabricating versatile cellulose films. Metal-organic frameworks (MOFs), as emerging nanomaterials, have been widely utilized in many fields such as catalysis [19], luminescence [20], adsorbents [21] and gas separation [22] due to their small size, controlled porosity, large surface area, and tunable multifunction. Meanwhile, the organic linker ligands or metal ions coming from MOFs can show flame retardant and UV blocking properties. For example, Zhou et al. [23] fabricated cellulose aerogels modified by Al-MIL-53. The existence of Al-MIL-53 endued the cellulose aerogels with excellent flame retardancy. In another study, Nabipour et al. [24] designed ZIF-8@cellulose composite aerogels through in situ synthesis strategy. The UL-94 vertical burning test result showed good self-extinguishing behavior of ZIF-8@cellulose composite aerogel. Many studies have focused on the use of MOFs for UV shielding. Studies on the use of nano-MOFs for imparting blue light shielding properties to cellulose materials have not been reported. For example, Zhang et al. [25] prepared an efficient UV blocking cotton fabric via the deposition of Cu-BTC, ZIF-8, and ZIF-67 on the fabric surface. Emam and Abdelhameed [26] fabricated UV-blocking textiles by embedding MIL-125(Ti)-NH_2_ nanoparticles (MNPs) into natural textiles by means of the one-pot method. However, to the best of our knowledge, the incorporation of nano-MOFs into a cellulose film to improve its flame retardant, mechanical strength and UV-blue light blocking properties, and maintain its nature properties such as flexibility and transparency has not been thoroughly studied.

In this work, we propose a facile concept of improving the mechanical, fire-resistant and ultraviolet (UV)-blue light shielding properties of cellulose-based composite bioplastics though in situ embedding MNPs into porous regenerated CH. In this concept, three-dimensional nanoporous CH acts as a nanoreactor and stabilizer for non-aggregate in situ growth and anchorage of MNPs. Then, hot-pressing leads to structural densification and generates transparent multifunctional MIL-125(Ti)-NH_2_@cellulose-based bioplastics (MNP@CBPs). To demonstrate the applicability of the concept, the influence of MNPs in improving mechanical, fire-retardant and ultraviolet-blue light blocking properties of MNP@CBPs was examined. Meanwhile, strong resistance of MNP@CBPs to penetration by aqueous/nonaqueous liquids was identified. Potential applications of MNP@CBPs would involve packaging materials for reducing food spoilage, screen protectors for eliminating harmful light, and promising candidates for next-generation sustainable and protective plastic products, among others.

## 2. Experiment

### 2.1. Materials and Reagents

Filter paper pulp (FP) with an ash content of 0.01% was purchased from Hangzhou Special Paper Industry Co., Ltd. (Hangzhou, China). Titanium isopropoxide (TiOiPr, 99%) was purchased from Aladdin Biochemical Technology Co., Ltd. (Shanghai, China). 2-aminoterephthalic acid (NH_2_-BDC, 99%) was obtained from Sigma-Aldrich. (Shanghai, China). Lithium hydroxide (LiOH) and urea were purchased from Shanghai Chemical Reagent Co., Ltd. (Shanghai, China). Rhodamine B (RhB) and titanium oxide (TiO_2_, 25 nm) were also obtained from Aladdin Industrial Corporation. Glycerol, *N*,*N-*dimethylformamide (DMF, 99.9%), anhydrous ethanol and methanol were all analytical grade, unless stated elsewhere.

### 2.2. Synthesis of Cellulose Hydrogel

FP was ground with an A11 basic grinder (IKA, Germany) to obtain cellulose powder. In a typical procedure, cellulose powder was added in an aqueous 4.6 wt% LiOH/15 wt% urea and stirred for 5 min at room temperature. The solution was used to dissolve cellulose at −20 °C in a refrigerator within 12 h. Subsequently, the mixture was thawed into an ice-water mixture at room temperature and the fibers were completely dissolved under vigorous stirring to obtain 4 wt% cellulose solution. The resultant viscous cellulose solution was subjected to ice water bath sonication for 20 min to remove air bubbles. Then, the resultant cellulose solution was cast on a glass mold, anhydrous ethanol was used to induce rapid regeneration, and then thorough washing with water was performed to form a cellulose hydrogel, which had a thickness of ca. 2.5 mm.

### 2.3. Synthesis of MIL-125(Ti)-NH_2_@Cellulose Hydrogels (MNP@CHs)

MNPs were synthesized at room temperature according to previous reports [26,27]. The cellulose hydrogels were soaked in anhydrous methanol several times to completely remove the water. A given amount of TiOiPr (see Table S1) was added to 60 mL of anhydrous methanol with vigorous stirring, and then sealed and placed in a 65 °C water bath to promote complete dissolution of titanium isopropoxide. The cellulose gels were added to the above solution and soaked for 1 h at 65 °C. A given amount of NH_2_-BDC (see Table S1) was dissolved in 24 mL of DMF and correspondingly added to a precursor solution containing cellulose gel. The mixture was then reacted at room temperature for 48 h. The MIL-125(Ti)-NH_2_@cellulose hydrogels (MNP@CHs) were washed with DMF to remove the remnant chemicals and washed with water to remove DMF. Depending on the content of MNPs in cellulose hydrogel, the MNP@CHs were labeled as MNP@CH1, MNP@CH2, MNP@CH3, MNP@CH4 and MNP@CH5. The amounts of MNPs in CHs were calculated for all modified samples. By immersing known weights of dry samples (freeze-drying) in 10 mL of dimethyl sulfoxide that contained 2 mL of hydrochloric acid (HCl, 35%), the samples were sonicated for the complete dissolution of MNPs. Samples were then removed, washed with deionized water several times, and then dried in vacuum. Sample weight before and after isolation of MNPs was recorded, and the differences in weight were assigned to the content of MNPs.

### 2.4. Synthesis of MIL-125(Ti)-NH_2_@Cellulose Bioplastics (MNP@CBPs)

The MNP@CHs were immersed in glycerin solution (5 g of glycerin dissolved in 95 g of water) for 30 min. Then, MNP@CHs were sandwiched between stainless steel plates and then dried at 110 °C for approximately 90 min at an applied pressure of about 0.1 MPa initially, and finally were hot pressed at an applied pressure of about 60 MPa for 30 min with an R32022015 hot press machine (Wuhan Qien Science & Development Co., Ltd., Wuhan, China). Thus, MNP@CBPs with a thickness of 0.25 mm were obtained, coded as MNP@CBP1, MNP@CBP2, MNP@CBP3, MNP@CBP4 and MNP@CBP5. For comparison purposes, a neat cellulose bioplastic (CBP) with thickness of about 0.25 mm was obtained using the same process.

### 2.5. UV-Shielding Measurement of MNP@CBPs

The UV-blocking performance of neat CBP and MNP@CBPs was evaluated by photocatalytic degradation of RhB solution using TiO_2_ NPs. The degradation behavior of RhB solution in the presence of TiO_2_ under UV light (250 W) was performed to assess the UV-shielding capability of MNP@CBPs. In short, 50 mg of TiO_2_ NPs and 100 mL of RhB solution (1 × 10^–5^ M) were mixed for complete dispersion. Before the photocatalytic experiment, the mixed solution was stirred in the dark for 30 min at 25 °C to reach adsorption/desorption equilibrium. Prior to the UV irradiation, the mouth of the beaker was covered with CBP or MNP@CBPs. The distance between the light source and the sheet was about 10 cm. At given intervals (t), 5 mL of the mixed solution was centrifuged to remove the TiO_2_ NPs. The absorbance of RhB at 552 nm was measured by a TU-1950 UV-vis spectrophotometer. The UV-blocking ability was calculated as *I* = *A_t_*/*A*_0_ × 100%, where *A*_0_ is the initial absorbance of RhB solution and *A_t_* is the absorbance of the RhB solution under UV radiation.

### 2.6. Characterizations

X-ray photoelectron spectroscopy (XPS) test was carried out with an ESCALAB 250Xi using Al Kα radiation. Fourier transform infrared (FTIR) spectra was collected on a FTIR spectrometer (Nicolet 6700, Thermo Fisher Scientific Inc., Waltham, MA, USA) in the frequency range of 4000–500 cm^−1^. A D/max 2200PC X-ray diffractometer (Rigaku Corporation, Tokyo, Japan) with Cu Ka radiation (*λ* = 0.154 nm) was used to collect the X-ray diffraction (XRD) patterns. For FTIR, XRD and XPS measurement, samples needed to be ground into powder with an A11 basic grinder (IKA, Deutschland, Germany). The scanning electron microscope (SEM, Hitachi S4800, Tokyo, Japan, operated at 3.0 kV) was used to identify the cross-sectional morphologies, and surface elemental composition analysis was conducted using energy-dispersive X-ray spectroscopy (EDS). The samples were frozen in liquid nitrogen, and snapped immediately. The samples were fixed on the sample platform, and then coated with platinum. Finally, SEM observations were conducted at different magnifications. Thermal gravimetric analysis (TGA) was performed using a STA449 F3 device in a nitrogen atmosphere at a heating rate of 10 °C·min^−1^. The surface topography of samples was characterized with a Multimode 8 Atomic Force Microscope (AFM, Bruker, Billerica, MA, USA), and images were collected. TEM images of samples were acquired with a transmission electron microscope (JEM-1400Plus, JEOL, Tokyo, Japan) at an accelerating voltage of 200 kV. Samples were embedded in an epoxy resin, and ultrathin slices were obtained for TEM measurements by sectioning on an LKB-8800 ultratome. The flame retardancy of samples was determined in terms of the oxygen index (OI), which was measured on a FTT0077 Oxygen Index Meter. The tensile stress–strain curves were mapped at an ambient temperature using a UTM2203 universal testing machine (Shenzhen SUNS Technology Stock Co. Ltd., Shenzhen, China) according to ISO527-3-1995 (E) at a speed of 2 mm min^–1^. Rectangular strips with 5 × 50 mm^2^ dimensions were used in tensile tests. The average values and standard deviations of the mechanical performances were evaluated by at least five specimens. The optical transmittance was tested using a UV-Vis spectrophotometer (TU-1950, Beijing, China). The photostability of samples was studied by analyzing UV spectra under the continuous exposure of UV irradiation with a 300 W ultraviolet lamp at 365 nm. The water contact angles of samples were performed by a Data Physics Instrument (Drop shape analysis system DSA-100/10, Kruss) in dynamic mode. Neat CBP and MNP@CBP, equipped as filters, were combined with glass filtration sets. A grease solution (mixture of castor oil, toluene, and *n*-heptane), colored ethanol and water were used to interact with CBP and MNP@CBP sheet for 12 h. Digital photographs were taken to assess liquid-barrier properties. After hot-press drying, the UV-Vis transmittance of MNP@CBP was measured.

## 3. Results and Discussion

### 3.1. Structure and Morphology of MNP@CBP

**Figure 1:**
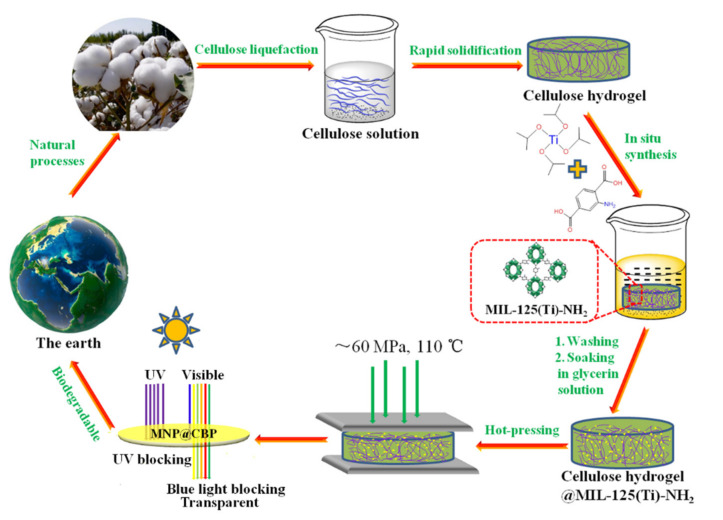
Schematic demonstration of the preparation process for MNP@CBP.

**Figure 2:**
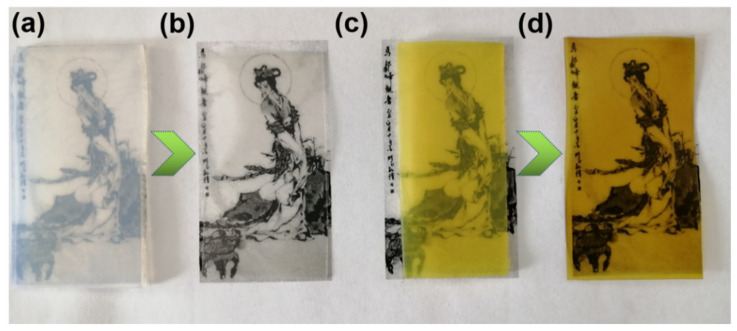
Digital photographs of samples placed on black pattern strip with printed “Chinese ink painting”. (**a**) CH, (**b**) CBP, (**c**) MNP@CH and (**d**) MNP@CBP.

Figure 1 and Supplementary Materials Figure S1 show the proposed process for the fabrication of MNP@CBP. Initially, the synthesis process involves the preparation of a homogeneous cellulose/LiOH/urea aqueous solution according to preliminary work [28], followed by physical gelation with anhydrous ethanol and washed with water to form a cellulose hydrogel. The photograph in Figure 2a shows that the cellulose hydrogel with about 2.5 mm thickness is transparent. Subsequently, MNPs are in situ loaded into the regenerated cellulose gel to obtain MNP@CH. The MNP@CH with approximately 2.5 mm thickness is also transparent (Figure 2c). The hot-pressing not only evaporated and removed the water rapidly from the cellulose hydrogel, but also changed the orientation and crystalline structure of the cellulose, leading to the structural densification [29]. One of the crucial criteria for plastics is the transition of their aggregation structure. In Figure 2b, the obtained CBP with about 0.25 mm thickness is transparent and shows a relatively high transmittance. Importantly, it is evident in Figure S2 in the Supplementary Materials that the cellulose microfibrils show a parallel arrangement, demonstrating that cellulose hydrogel composed of cellulose molecular chains was transferred into transparent CBP after hot pressing. The MNP@CBP, despite containing a metal-organic framework, still has good transparency after hot-pressing (Figure 2d).

**Figure 3:**
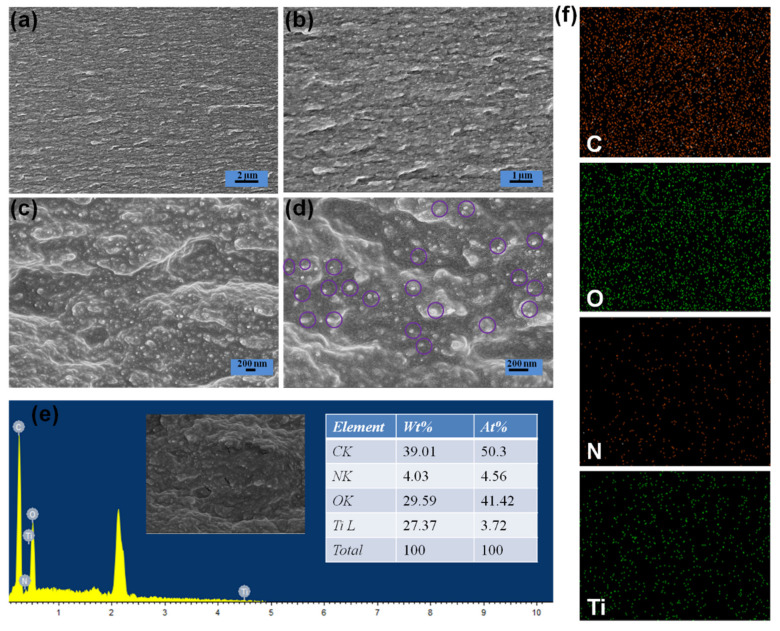
(**a**–**d**) Cross-sectional SEM images of MNP@CBP5; (**e**) EDS spectrum of MNP@CBP5; (**f**) elemental mapping images of MNP@CBP5 for carbon, oxygen, nitrogen and titanium. × 5000 magnification (**a**), ×10,000 magnification (**b**), ×50,000 magnification (**c**), ×100,000 magnification.

To observe the morphology of the MNP@CBP filled with MNPs, representative cross-sectional SEM of an MNP@CBP5 was observed. In Figure 3a,b, the SEM images of the MNP@CBP5 show the same stacked structure as the pure CBP and the MNPs are tightly stacked in the MNP@CBP5 with magnified SEM images, the MNPs (at the circled) are well-distributed in the MNP@CBP5 without congregation (Figure 3d). For MNPs, particles with size distribution of 40.4–55.6 nm in MNP@CBP5 was detected. MNPs are firmly embedded in the cellulose matrix, which indicates a strong interfacial adhesion between cellulose and MNPs. Such a homogeneous dispersion and alignment of MNPs, as well as a favorable interface, are beneficial to improving the UV-blue light blocking and mechanical properties of the MNP@CBPs. EDS analysis also confirmed the formation of MNPs inside MNP@CBP5 (Figure 3e). Signals of nitrogen and titanium were recorded in EDS analysis patterns for cellulose decorated by MNPs. Elemental mapping images (Figure 3f) of MNP@CBP also revealed the uniform distribution of MNPs, as demonstrated by the uniform detection of titanium and nitrogen atoms in MNP@CBP5 apart from the carbon and oxygen atoms. To further prove the successful synthesis of MNPs in MNP@CBPs, TEM images of MNP@CBP5 were observed (Supplementary Materials Figure S3). A uniform dispersion of MNPs was observed.

The surface morphology of the pure CBP and MNP@CBP is studied using AFM (Supplementary Materials Figure S4). As a reference, the AFM image of neat CBP clearly displays a homogeneous and flat surface (Supplementary Materials Figure S4a). Meanwhile, it can be obviously seen that, with the incorporation of MNPs, the surface of the MNP@CBP5 is still flat (Supplementary Materials Figure S4b), which shows that the presence of MNPs does not affect the smoothness of the material structure. This is mainly because MNPs are mainly synthesized inside the gel and the hot-pressing process also causes the surface of the MNP@CBP to be smooth. The roughness average (Ra) of pure CBP and MNP@CBP5 was about 8.6 and 9.12 nm, respectively.

### 3.2. FTIR Analysis

**Figure 4:**
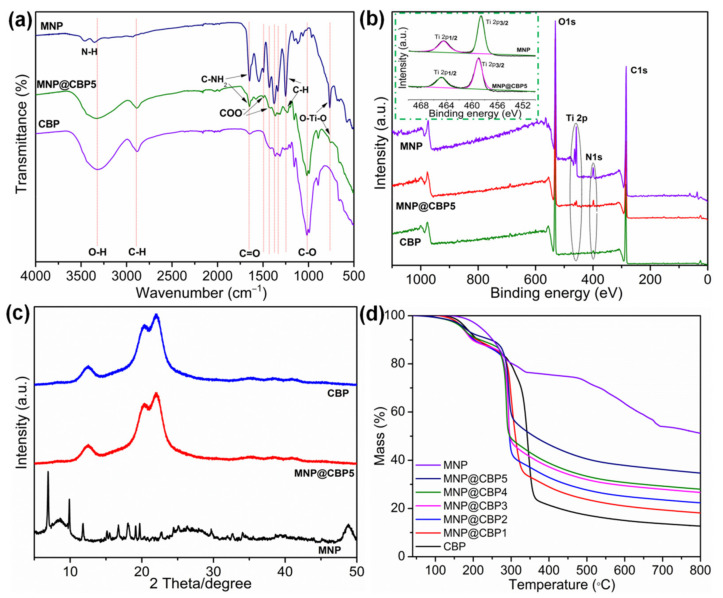
(**a**) FTIR spectra of CBP, MNP and MNP@CBP5 powders; (**b**) the full wide-scan XPS spectra of CBP, MNP and MNP@CBP5 powders, and the high resolutions Ti 2p spectra of MNP and MNP@CBP5 powders; (**c**) XRD patterns of CBP, MNP and MNP@CBP5 powders; (**d**) thermogravimetric analysis of CBP, MNP and MNP@CBPs powders.

FTIR spectra of neat CBP, MNPs and MNP@CBP5 are shown in Figure 4a. Before the test, pure CBP and MNP@CBP were ground into powder. For pure CBP, the characteristic peaks at 3321 and 2888 cm^−1^ were attributed to the OH and C–H stretching vibrations of the sugar ring, respectively. The peaks at 1644 cm^−1^ were assigned to the stretching vibration of C=O. The peak at 1021 cm^−1^ was assigned to the stretching vibration of C–O. For MNP powder, the peaks at 1652, 1545, 1431 and 1373 cm^−1^ are attributed to asymmetric and symmetric carbonyl stretching vibrations in MNPs, while the peaks at 1250 cm^−1^ assigned to the C–H symmetric stretching vibrations of the benzene ring. Moreover, peaks range from 400 to 800 cm^−1^ shows the Ti–O–Ti–O vibrations [30]. The peaks at 3358 and 3464 cm^−1^ were attributed to amino groups in backbone of MNPs. After the introduction of MNPs, the appearance of a new peak at 755 cm^−1^ was attributed to the vibration of O–Ti–O in MNP@CBP [26,31]. Furthermore, two new peaks appeared at 1496 and 1431 cm^−1^ after loading MNPs, attributed to the COO^−^ vibrational modes from the MOF linker [26], demonstrating successful synthesis of MNPs in MNP@CBPs.

### 3.3. XPS Analysis

To further confirm that MNPs formed inside MNP@CBPs, XPS was used to confirm the chemical composition of MNP@CBP5 powder. In Figure 4b, the survey scan showed that CBP consists of (β→4) linked D-glucose units containing only carbon (C 1s), oxygen (O 1s) peaks at 284.8 and 533.1 eV, and no titanium (Ti 2p) and nitrogen (N 1s) signal peaks. In the MNPs, the main strong Ti 2p, C 1s, O 1s and N 1s signal peaks were present at around 458.7, 284.8, 533.1 and 399.6 eV, respectively, suggesting C, Ti, O and N four elements existed on MNPs. In MNP@CBP5, two new peaks appear at 458.7 and 399.6 eV corresponding to Ti 2p and N 1s, respectively, demonstrating the existence of N and Ti elements, which also matched well with obtained EDS results. This proved that MNPs were successfully synthesized in MNP@CBP. In the Ti 2p core level spectrum of MNP@CBP5, symmetric peaks for Ti 2p_1/2_ and Ti 2p_3/2_ appeared at 464.5 and 458.8 eV, respectively, which indicates the existence of a normal state of titanium IV bounded to oxygen for the titanium-oxo cluster, similarly to the pure MNPs [32]. The XPS analysis can identify that MNPs are successfully formed in MNP@CBP5.

### 3.4. XRD Analysis

Figure 4c shows that the ray diffraction spectrum of neat CBP has three strong diffraction peaks at 12.4°, 20.2°, and 22.2°, corresponding to the diffraction of the (110), (1Î0) and (200) faces of the cellulose II type, demonstrating that the crystal structure of the sample did not change, showing the cellulose type II crystal structure [33]. For MNPs, all peaks are in accordance with previous reports [26,34]. The XRD patterns of MNP@CBP5 display no distinct MNP-characteristic peaks, only the cellulose diffraction peaks, which may be attributed to two reasons. One is the low content of MNPs in MNP@CBP5, and the other is that MNPs are firmly embedded in MNP@CBP5, so that fewer MNPs are exposed after grinding.

### 3.5. TGA Analysis

The thermal stability of pure CBP, MNPs and MNP@CBPs was investigated by thermogravimetric analysis, and the respective curves were displayed in Figure 4d. The MNPs show a gradual 24.2% mass loss up to 338 °C, which can be ascribed to the guest molecules removal from the cavities, the evaporation of H_2_O molecules in the frameworks or the elimination of non-reacted organic ligands from the MNPs’ surfaces. The second weight loss step (~23%) beginning at ~470 °C is owing to the structural decomposition of MNPs [35]. The weight of the final residue was ~49.3%. The pure CBP exhibits a three-step thermal degradation behavior: the first step, corresponding to a 16% weight loss, occurs in the range of 40–275 °C, which is attributed to the elimination of residual moisture from the specimens. The subsequent 60% mass loss occurs in the range of 280–370 °C, and is a sign of the carbohydrate polymers’ degradation. The final 10% mass loss at 370–800 °C could be ascribed to the oxidation of the residues, leaving a small amount of residue (12 wt%) at 800 °C. As with neat CBP, the decomposition of all MNP@CBPs also involves three steps. It can be seen that the thermal stability of all the MNP@CBPs is increased, and they have higher amounts of the residues. The enhancement of thermal stability could be owing to the existence of MNPs, as they efficiently inhibit the volatilization of the decomposition products into the gas phase. The residue fractions of all the MNP@CBPs at 800 °C are higher than the neat CBP. This indicates that the addition of MNPs significantly improves the thermal stability of MNP@CBPs.

### 3.6. Suggested Mechanism of Interaction

**Figure 5:**
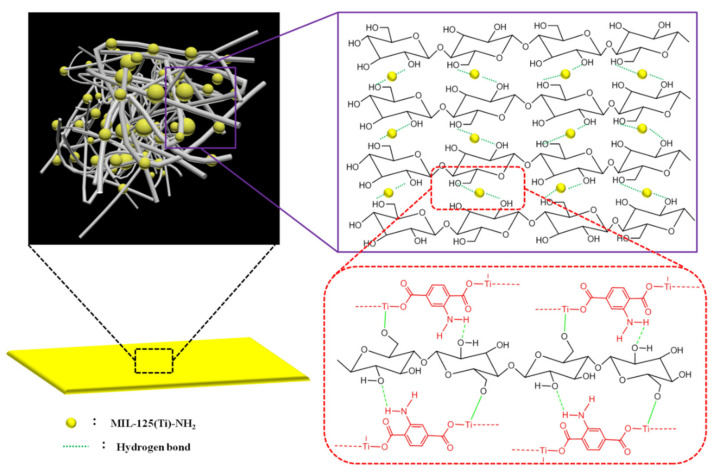
Schematic of the intermolecular hydrogen bonding between cellulose and MNPs in MNP@CBP.

The in situ formation of MNPs within MNP@CBPs was confirmed via the results of SEM micrographs, FTIR and XPS spectra. The interaction mechanism between MNPs and cellulose was proposed and presented in Figure 5. Cellulosic material contains a larger number of active sites represented in hydroxyl groups, which offered a good way to interact with Ti of MNPs by complexation. During the formation of MNPs, cellulose was supposed to act as a chelator in cooperation with 2-aminoterephthalic acid and the formed complex with metal ions through its hydroxyl groups. After the formation of MNPs, cellulose was argued to interact chemically with MNPs via the formation of hydrogen bonding and coordination linkage between the amine groups and metal central ion of MNPs with the functional groups of cellulose [36,37,38]. After hot-press drying, the MNPs were further firmly fixed inside the MNP@CBP.

### 3.7. UV-Blue Light Resistance of MNP@CBPs

**Figure 6:**
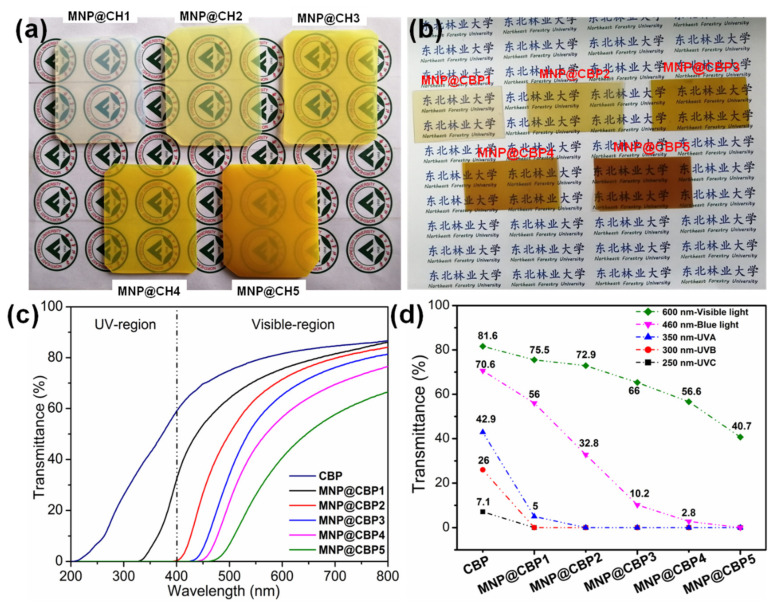
(**a**) Photographs of MNP@CHs, (**b**) photographs of MNP@CBPs, (**c**) UV-vis light transmittance curves of CBP and MNP@CBPs from 200 to 800 nm, (**d**) transmittance at specific UV light and blue light wavelengths.

In Figure 6a,b, the MNP@CHs and MNP@CBPs have a yellow color owing to the existence of MNPs, and the color of MNP@CHs and MNP@CBPs will gradually deepen, which is due to the fact that with the increase in the concentration of reaction reagent, the amount of MNPs formed in the cellulose hydrogel also increases (Supplementary Materials Figure S5). The UV-vis transmittance of the CBP and MNP@CBPs was measured in the wavelength range 200–800 nm. Opaqueness of cellulose-based film materials is known to be due to microsized cavities within the fibrous network, which cause strong light scattering and thus the light transmittance [39,40]. Hence, a cellulose film with a compact inner structure will have a greatly improved optical performance. It can be seen from Figure 6c,d, due to the well-aligned and densely packed mode of fibers (Supplementary Materials Figure S2), that pure CBP can effectively avoid light scattering and exhibit high optical transmittance in the visible light region (81.6% at 600 nm). Unfortunately, the pure CBP has a poor shielding effect from UV and blue light. After incorporating MNPs, the MNP@CBPs exhibit an attractive UV-blue light blocking capacity. The results show that as the content of MNsP increases, the UV-blue light in the range of 200–500 nm is gradually blocked, while the transparency gradually decreases at 600 nm. The MNP@CBP1 can almost block the whole UVB region below 320 nm, which shows that even a very low content of MNPs can effectively block UV light. Meanwhile, MNP@CBP1 showed a high transparency of about 75.5% at 600 nm. As the content of MNPs increases, 100% of UVB light and UVA light are shielded by MNP@CBP2, whereas the transparency deceases (72.9% at 600 nm). This indicates that the MNP@CBP2 showed almost complete UV-shielding covering both regions of UV-C (200–280 nm), UV-A (280–320 nm) and UV-B (320–400 nm). The MNP@CBP3 possessed good visible-light transparence of 66% at 600 nm and superior UV-blue light blocking ability (100% UV and nearly 69% blue light). Importantly, it can be seen that the MNP@CBP3 basically filters out > 94% harmful blue light (below 455 nm). Although the MNP@CBP4 and MNP@CBP5 have high UV-blue light shielding capability, the transparency is relatively low.

**Figure 7:**
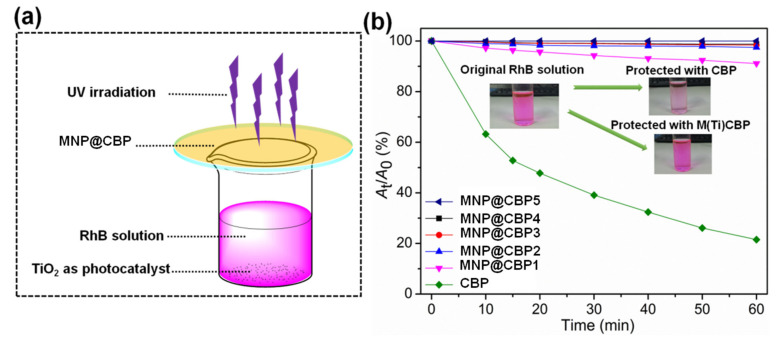
(**a**) Experimental schematic diagram of photodegradation of RhB based on MNP@CBP. (**b**) Photodegradation curves of RhB solutions protected by MNP@CBPs. The inserted photographs: the original RhB solution, and the solution protected with neat CBP and MNP@CBP, respectively.

In order to further examine the UV-shielding performance of MNP@CBPs, a RhB solution protected with neat CBP or MNP@CBPs was exposed to UV light, and the decay curves of the absorption intensity of the RhB solution at 552 nm were examined. The UV shielding experiment is shown in Figure 7a. In Figure 7b, the RhB solution protected with neat CBP displayed a significant degradation, which reached 68.5% after UV irradiation for 60 min. When MNP@CBP1 was used as a shielding material, the degradation rate of RhB decreased significantly. The RhB solution shielded by MNP@CBP1 shows a decrease of 9%. When MNP@CBP2 served as the shielding material, only about 2.5% of RhB degraded. With increasing MNP content, i.e., using MNP@CBP3, MNP@CBP4 and MNP@CBP5 as shielding materials, there was almost no degradation of RhB, with 1.6%, 1.2% and 0.5% degradation rates, respectively, demonstrating the outstanding UV-shielding ability of MNP@CBPs. In addition, compared to the original RhB, the solution protected with neat CBP showed an obvious discoloration due to the large photodegradation of RhB, whereas that protected with MNP@CBP5 displayed a slight change after UV irradiation and was similar to the original color of RhB. The results reveal that MNP@CBP had excellent UV-shielding performance.

**Figure 8:**
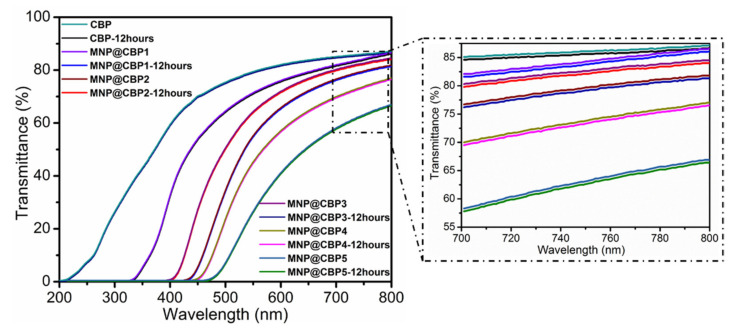
Photostability of CBP and MNP@CBPs.

The service lifetime of UV-shielding materials is of significant concern. Consequently, the photostability is a key factor that affects the long-term application of UV protection materials. In order to evaluate the photostability of MNP@CBPs, the as-prepared MNP@CBPs were irradiated by a continuous photoexcitation at 365 nm for 12 h before the test. Figure 8 shows UV-vis transmittance curves of CBP and MNP@CBPs before and after continuous UV treatment for 12 h. The results show that the transmittance of pure CBP presents a stable state under UV irradiation, which implies that the pure CBP has good photostability. After 12 h of UV irradiation, no obvious change can be observed in transmittance spectra for all the MNP@CBPs, indicating that the irradiated MNP@CBPs can still maintain the original transparency and UV-blocking ability. This result demonstrates the high photostability of MNP@CBPs.

**Figure 9:**
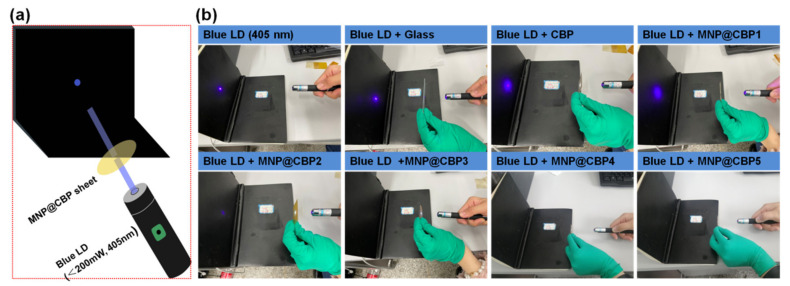
(**a**) Scheme of the experimental setup, (**b**) photographs of blue LD and its blocking effect covered by glass, CBP and MNP@CBPs.

The visual blue light shielding experiment is shown in Figure 9a. Figure 9b shows the photographs of glass, neat CBP and MNP@CBPs used to shield blue laser diode (LD, 405 nm). In the case of glass and CBP, the intensity of transmitted blue light is similar to that of blue LD, which indicates that glass and pure CBP have no blue light shielding capability. For the MNP@CBPs, the intensity of the transmitted blue light gradually diminished with increasing MNP loading, which can be seen with the naked eye. In particular, blue light can be completely blocked by using the MNP@CBP3, MNP@CBP4 and MNP@CBP5.

**Figure 10:**
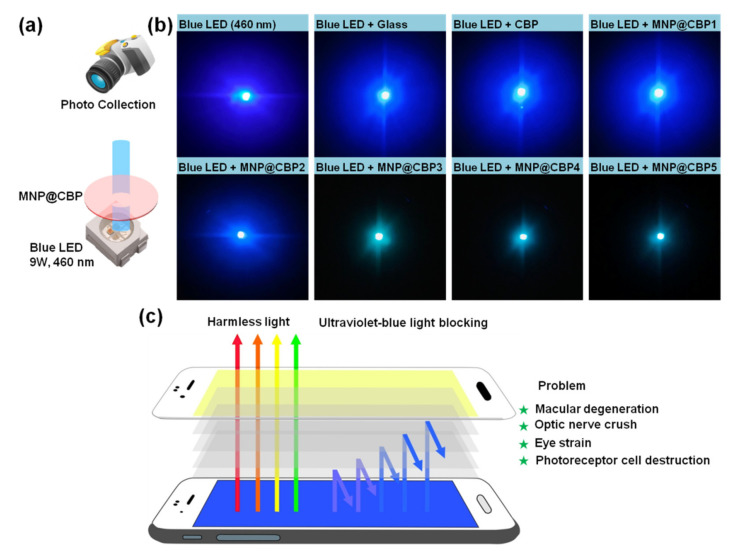
(**a**) Schematic of the experimental setup, (**b**) photographs of blue LED and its blocking effect covered by glass, CBP and MNP@CBPs. (**c**) Schematic diagram of MNP@CBP applied in a mobile phone screen protector.

To further investigate the applicability of the MNP@CBPs as blue light blocking materials, CBP and MNP@CBPs are used to shield a blue InGaN LED chip (blue LED, 460 nm). The experiment setup is shown in Figure 10a. Figure 10b shows photographs of the glass, CBP and MNP@CBPs on the blue LED. The photographs of the glass and CBP on the blue LED show that the visible intensity of the blue light is almost unchanged. In the case of the MNP@CBP3, MNP@CBP4 and MNP@CBP5, the blue light intensity of the InGaN LED is significantly weaker than that of the glass, CBP, MNP@CBP1 and MNP@CBP2. Figure 10c shows the application of MNP@CBP in a mobile phone screen protector, which can prevent eye damage caused by electronic devices.

### 3.8. Flame Retardant Property

**Figure 11:**
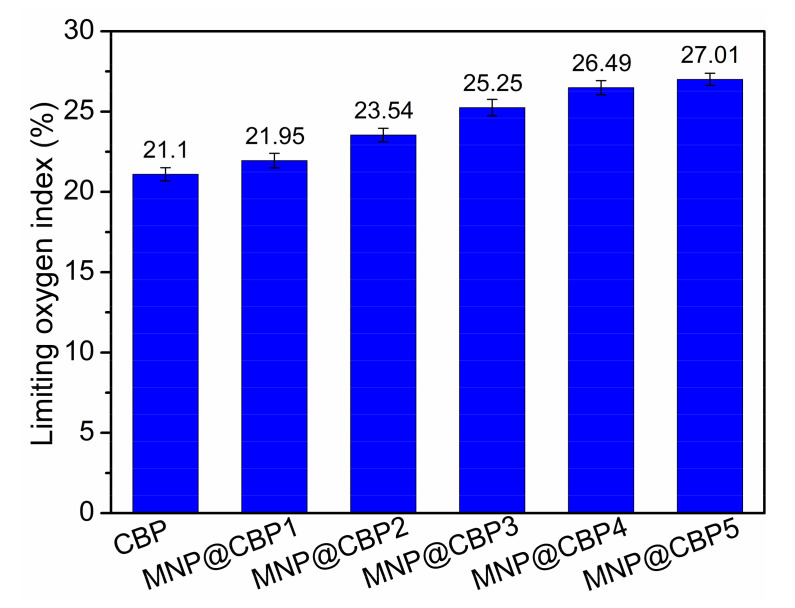
Limiting oxygen index of CBP and MNP@CBPs.

The flame retardant property is critical for applications of cellulosic materials, so the limited oxygen index was adopted to assess the flame retardance of MNP@CBPs. Figure 11 shows the LOI values of the MNP@CBP with different MNP contents. Evidently, the incorporation of MNPs into the cellulose matrix causes a significant increase in LOI values of MNP@CBPs. The neat CBP had a LOI value of only 21.1%, indicative of an easily flammable material in nature. The LOI value of the MNP@CBPs increased from 21.95 to 27.01%. The results show that MNPs can improve the flame retardancy of MNP@CBPs. In Supplementary Materials Figure S6, when the flame was applied, the CBP and MNP@CBP1 immediately ignited and burned with a flame that lasted throughout the test. In contrast, once the flame was applied to the MNP@CBP2-MNP@CBP5, it burned very slowly and self-extinguished as soon as the flame was removed from the samples. This result also shows that the introduction of MNPs is beneficial in improving the flame retardancy of the material.

### 3.9. Mechanical Property

**Figure 12:**
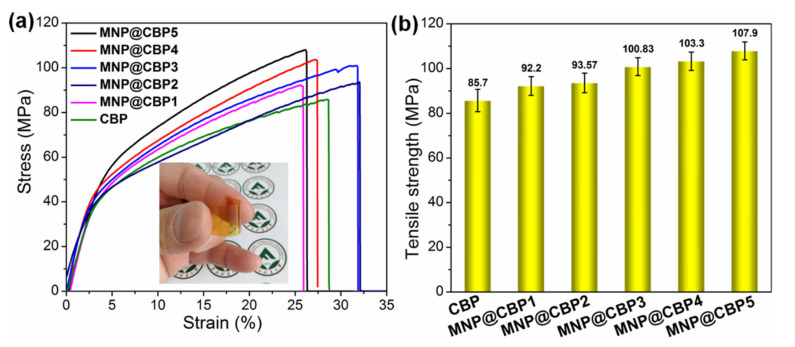
(**a**) Representative stress–strain curves of CBP and MNP@CBPs, (**b**) tensile strength of CBP and MNP@CBPs. The inset is a digital image of the MNP@CBP to show good mechanical robustness and flexibility.

Mechanical strength is another crucial parameter for practical application of cellulose-based film materials. Figure 12 exhibits the stress–strain curve for neat CBP and MNP@CBPs. The CBP possesses a tensile strength of 85.7 MPa. As a result of dissolution, regeneration and hot pressing of cellulose hydrogel sheet, the formation of a more compact/closed structure with a drastically increased number of intermolecular bonds would explain the high mechanical robustness of CBP. As expected, the incorporation of MNPs into the cellulose matrix has a significant influence on the tensile deformation behavior of MNP@CBPs. Most attractively, the tensile strength of the MNP@CBPs show an apparently increasing trend, as depicted in Figure 12. For example, the tensile strength of MNP@CBP5 is 107.9 MPa. Compared with that of neat CBP, there is a 25.9% enhancement in tensile strength. This substantial increment in the tensile properties of MNP@CBPs can be ascribed to (i) compact structure and uniform distribution of MNPs in the cellulose matrix, as analyzed by SEM (Figure 2) and (ii) their good interaction by adequate hydrogen bonding interaction. Meanwhile, the MNP@CBP was also easy to handle and tolerated bending (Figure 10a).

### 3.10. Contact Angle and Barrier Property

**Figure 13:**
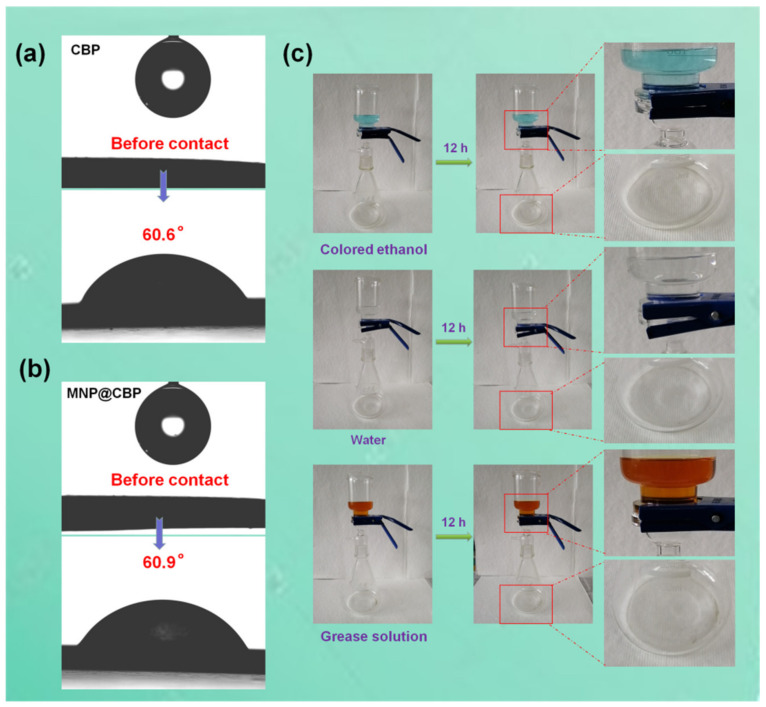
(**a**,**b**) Water contact angle images of the CBP and MNP@CBP3, (**c**) interaction between MNP@CBP3 and water, colored ethanol and grease solution (mixture of castor oil, toluene, and *n*-heptane, with volume percentages of 30%, 35%, and 35%, respectively).

High liquid-resistant properties of cellulosic paper/film are highly desirable for practical application. However, compared to fossil-fuel plastics which are derived from petroleum, applications of polysaccharide-based products are strongly hampered by limited resistance to penetration by liquids. The surface anchorage of barrier coatings is oftentimes applied to endow cellulosic products with liquid-barrier properties. A recently published interesting work [41] pertained to the anchorage of a regenerated cellulose coating (via in situ cross-linking) to filter paper, resulting in significant liquid-resistance as well as mechanical strength. As an alternative to the use of barrier coatings, structural reorganization was found to be very effective in the development of liquid-barrier properties. As displayed in Figure 13a,b, the water-contact angles for CBP and MNP@CBP are 60.6° and 60.9°, respectively. MNP@CBP has a strong resistance to penetration by colored water, colored ethanol and grease solution (Figure 13c). In Supplementary Materials Figure S7, MNP@CBP3 still has good UV-blue light blocking capability after 12 h of contact with water.

## 4. Conclusions

In this work, multifunctional bioplastics were successfully prepared through in situ embedding MNPs into regenerated cellulose gels then hot-pressing. Regenerated cellulose gel with a porous structure acts as a nanoreactor and stabilizer to facilitate growth and anchorage of MNPs. The SEM micrographs confirmed the uniform distribution of MNPs in the MNP@CBPs. The well-distributed MNPs can endow MNP@CBPs with sound UV-blue light shielding performance while retaining good optical transparency. Meanwhile, with the incorporation of MNPs, the mechanical strength of MNP@CBPs is increased by 6.5–25.9%. Furthermore, MNPs enhance the flame retardant effect of the MNP@CBPs. The limited oxygen index (LOI) of the MNP@CBPs increased from 21.95 to 27.01%. The hot-pressing technology improves the strong resistance of the MNP@CBPs to the penetration of water/non-aqueous liquids. All in all, by applying MNPs embedded into regenerated cellulose gel followed by hot pressing, we fabricated transparent functional bioplastics with significantly enhanced physical and functional properties suitable for applications in multiple fields such as transparent packaging, electronic devices and next-generation sustainable and protective plastic products.

## Data Availability

The authors confirm that the data supporting the findings of this study is available within the article.

## Supplementary Materials

The following are available online at https://www.mdpi.com/article/10.3390/polym13152433/s1, Figure S1: Fabrication of MIL-125(Ti)-NH_2_@cellulose bioplastic (MNP@CBP) from filter paper pulp, Figure S2: (a, b) Cross-sectional SEM images of CBP; (c) EDS spectrum of CBP; (d) Elemental mapping images of CBP for carbon and oxygen, Figure S3: TEM images of MNP@CBP5, Figure S4: AFM images of pure CBP (a) and MNP@CBP5 (b), Figure S5: Deposition ratio of MNP into MNP@CBPs, Figure S6: Photos of the samples before and after burning, Figure S7: UV transmittance curves of MNP@CBP3 after 12 hours of contact with water, Table S1: Reagents dosage, reaction time and temperature used for sample preparation.
