# Supplementary material for: Nano-Metal Organic Framework for Enhanced Mechanical, Flame Retardant and Ultraviolet-Blue Light Shielding Properties of Transparent Cellulose-Based Bioplastics
Source: Polymers (Basel). 2021 Jul 23;13(15):2433. doi: 10.3390/polym13152433 (PMC8348410; doi:10.3390/polym13152433)
Supplement: Supplementary file 1 [file polymers-13-02433-s001.zip › polymers-1259707-supplementary.pdf]

polymers

MDPI

Article

Nano-Metal Organic Framework for Enhanced Mechanical, Flame Retardant and Ultraviolet-Blue Light Shielding Properties of Transparent Cellulose-Based Bioplastics

Lijian Sun, Limei Li, Xianhui An and Xueren Qian*

Key Laboratory of Bio-Based Material Science & Technology Ministry of Education, Northeast Forestry University, Harbin 150040, China; lantian0308@ nefu.edu.cn (L.S.); limeili937@foxmail.com (L.L.); anxianh509@163.com (X.A.)

* Correspondence: qianxueren@nedu.edu.cn; Tel.: +86-13304642918

Citation: Sun, L.; Li, L.; An, X.; Qian, X. Nano-Metal Organic Framework for Enhanced Mechanical, Flame Retardant and Ultraviolet-Blue Light Shielding Properties of Transparent Cellulose-Based Bioplastics. *Polymers* **2021**, *13*, 2433. https://doi.org/10.3390/polym13152433

Academic Editor: Sergio Torres-Giner

Received: 29 May 2021
Accepted: 20 July 2021
Published: 23 July 2021

Publisher's Note: MDPI stays neutral with regard to jurisdictional claims in published maps and institutional affiliations.

Copyright: © 2021 by the authors. Licensee MDPI, Basel, Switzerland. This article is an open access article distributed under the terms and conditions of the Creative Commons Attribution (CC BY) license (http://creativecommons.org/licenses/by/4.0/).

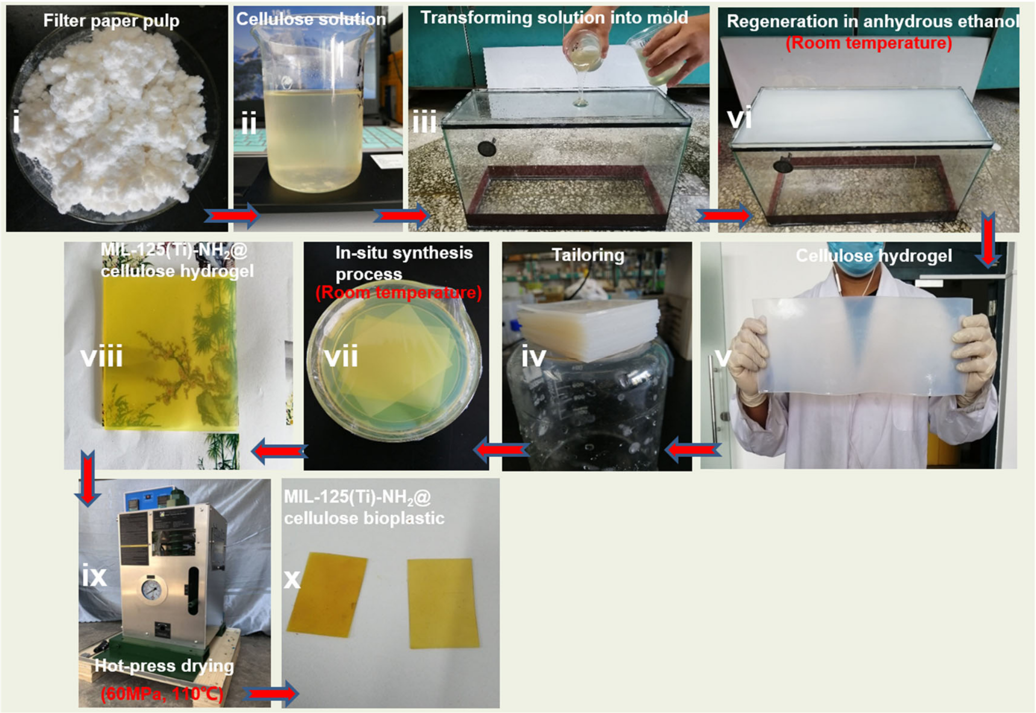

Figure S1. Fabrication of MIL-125(Ti)-NH$_2$@cellulose bioplastic (MNP@CBP) from filter paper pulp.

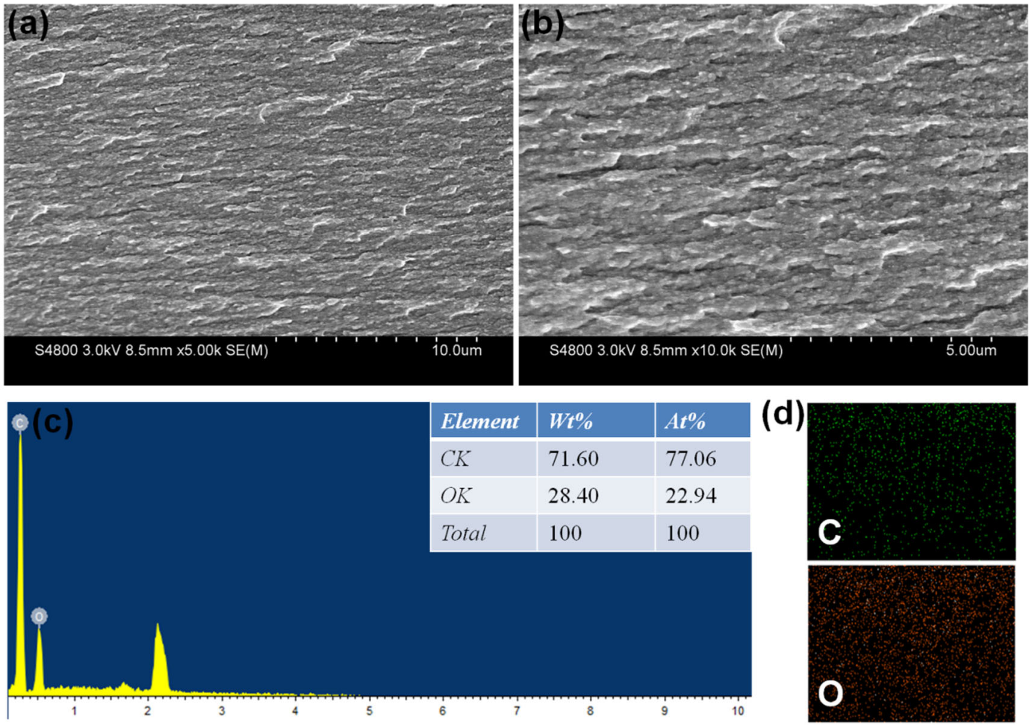

Element	Wt%	At%
CK	71.60	77.06
OK	28.40	22.94
Total	100	100

Figure S2. (a, b) Cross-sectional SEM images of CBP; (c) EDS spectrum of CBP; (d) Elemental mapping images of CBP for carbon and oxygen.

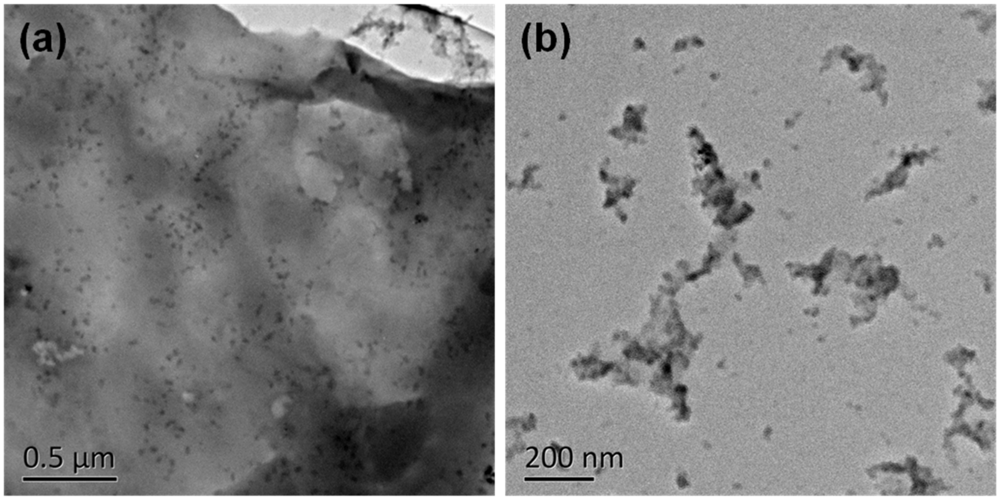

Figure S3. TEM images of MNP@CBP5.

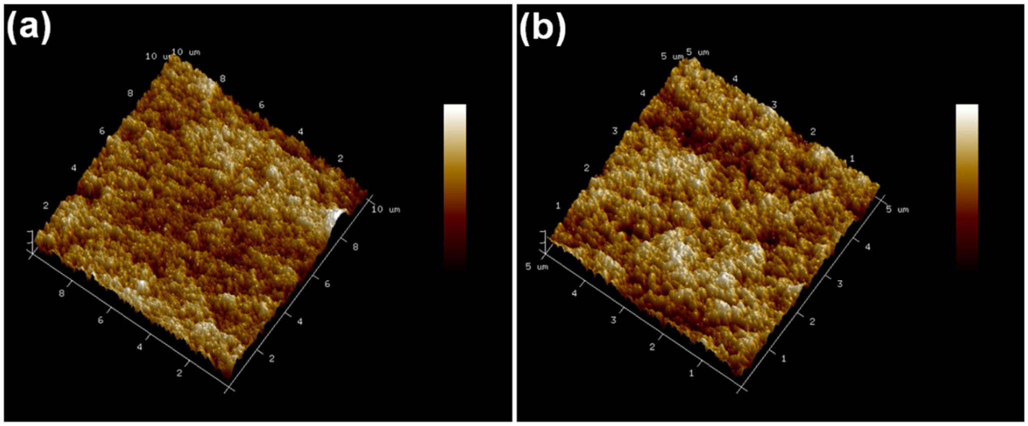

Figure S4. AFM images of pure CBP (a) and MNP@CBP5 (b).

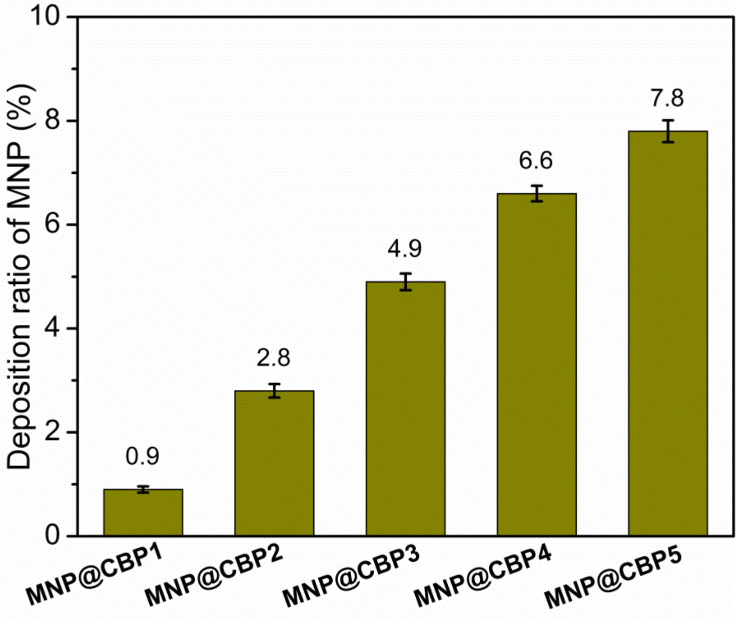

Figure S5. Deposition ratio of MNP into MNP@CBPs.

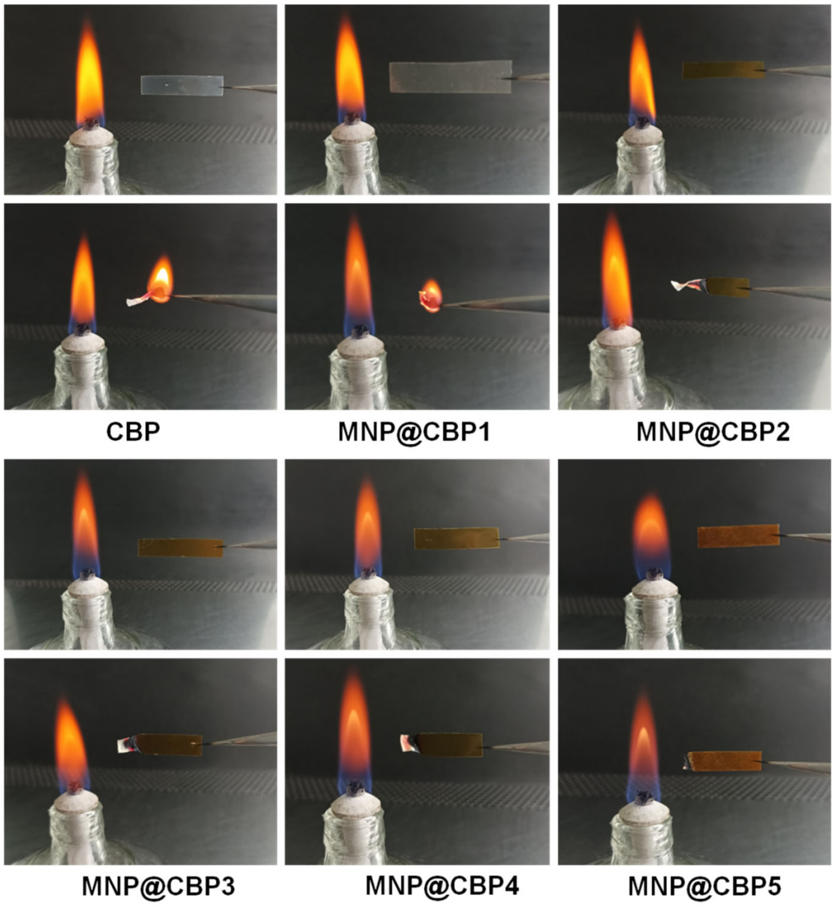

Figure S6. Photos of the samples before and after burning.

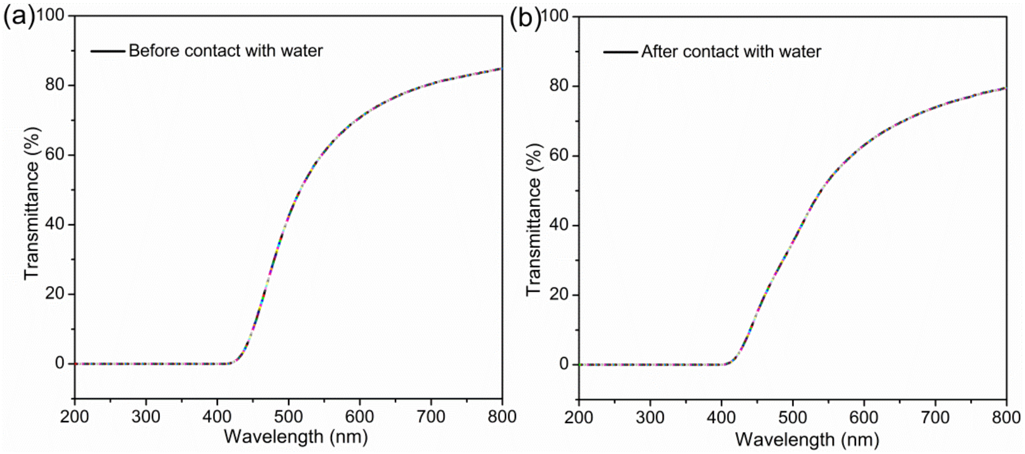

Figure S7. UV transmittance curves of MNP@CBP3 after 12 hours of contact with water.

Table S1. Reagents dosage, reaction time and temperature used for sample preparation.

**Sample**	**TiOiPr (mmol)**	**NH_2-BDC (mmol)**	**Time (h)**	**Temperature (°C)**
MNP@CBP1	0.335	0.55	48	25
MNP@CBP2	0.67	1.1	48	25
MNP@CBP3	1.34	2.2	48	25
MNP@CBP4	2.01	3.3	48	25
MNP@CBP5	2.68	4.4	48	25
